# Automated review of patient position in DIBH breast hybrid IMRT with EPID images

**DOI:** 10.1002/acm2.14038

**Published:** 2023-07-14

**Authors:** Jonathan Redekopp, Ryan Rivest, David Sasaki, Stephen Pistorius, Jorge E. Alpuche Aviles

**Affiliations:** ^1^ Department of Physics & Astronomy University of Manitoba Manitoba Winnipeg Canada; ^2^ Medical Physics Cancer Care Manitoba Manitoba Winnipeg Canada; ^3^ Department of Radiology University of Manitoba Manitoba Winnipeg Canada; ^4^ CancerCare Manitoba Research Institute Manitoba Winnipeg Canada

**Keywords:** automated chest wall tracking, breast RT, DIBH, EPID imaging, intrafraction motion, setup error

## Abstract

Deep Inspiration Breath Hold (DIBH) is a respiratory‐gating technique adopted in radiation therapy to lower cardiac irradiation. When performing DIBH treatments, it is important to have a monitoring system to ensure the patient's breath hold level is stable and reproducible at each fraction. In this retrospective study, we developed a system capable of monitoring DIBH breast treatments by utilizing cine EPID images taken during treatment. Setup error and intrafraction motion were measured for all fractions of 20 left‐sided breast patients. All patients were treated with a hybrid static‐IMRT technique, with EPID images from the static fields analyzed. Ten patients had open static fields and the other ten patients had static fields partially blocked with the multileaf collimator (MLC). Three image‐processing algorithms were evaluated on their ability to accurately measure the chest wall position (CWP) in EPID images. CWP measurements were recorded along a 61‐pixel region of interest centered along the midline of the image. The median and standard deviation of the CWP were recorded for each image. The algorithm showing the highest agreement with manual measurements was then used to calculate intrafraction motion and setup error. To measure intrafraction motion, the median CWP of the first EPID frame was compared with that of the subsequent EPID images of the treatment. The maximum difference was recorded as the intrafraction motion. The setup error was calculated as the difference in median CWP between the MV DRR and the first EPID image of the lateral tangential field. The results showed that the most accurate image‐processing algorithm can identify the chest wall within 1.2 mm on both EPID and MV DRR images, and measures intrafraction motion and setup errors within 1.4 mm.

## 1 INTRODUCTION

Breast cancer remains one of the cancers with significant mortality rates for women worldwide.^1^, ^2^ One of the treatment strategies for this disease consists of surgically removing the tumor followed by radiotherapy delivered to the affected breast.^3^ During breast radiotherapy, the heart may become irradiated, which is of particular concern for left‐sided breast patients.^4^, ^5^ Heart irradiation during treatment can increase the risk of a patient experiencing cardiac events later in life.^6^, ^7^

Deep Inspiration Breath Hold (DIBH) has been adopted in breast radiotherapy to lower cardiac irradiation.^8^, ^9^ It involves the patient taking in and holding a deep breath before the treatment beam is turned on, pushing the heart away from the path of the beam.^10^, ^11^ Cardiac doses have been reported to be significantly lowered with the use of the DIBH technique.^9^, ^12^, ^13^, ^14^, ^15^, ^16^ When performing DIBH, it is important to have a monitoring system in place to ensure the patient's breath hold remains stable and is reproducible at each fraction. Monitoring systems include 3D surface imaging systems, the alignment of lasers to patient skin tattoos, and a marker block placed on the patient's xiphoid process.^4^, ^8^, ^9^

Monitoring systems often rely on an external surrogate to infer the target's location and thus may not accurately track the location of the patient's internal anatomy. For example, a study by Conroy et al. evaluated DIBH using lasers to skin tattoo technique using 42 patients and EPID images. The study measured intrafraction motion, setup error, and interbeam (i.e., change between two different medial fields) motion.^8^ Their results showed that while the laser was aligned correctly to the patient's skin surface, for 19.4% of the treatment fields the maximum difference in chest wall position between the digitally reconstructed radiograph (DRR) and cine EPID frames was greater than the 5 mm setup tolerance.^8^ Additionally, 3.2% of patients exceeded the 3 mm tolerance for intrafraction/interbeam motion, with up to 6 mm of motion occurring while the laser remained aligned to the marker line drawn on the patient.^8^ Similarly, Lutz et al. performed a study with the Real‐time Position Management system to perform DIBH for 58 patients.^9^ Their results showed that, for 25 patients, the measured intrafraction motion exceeded the RPM's gating window.^9^ This shows that the position of the chest wall can deviate while the external surrogate position remains within tolerance.^9^ Laaksomaa et al. used a 3D surface‐imaging system for treating 50 patients, which was validated with a combination of kV and MV imaging.^10^ They found that when using only the surface imaging system for patient setup, where the patient's surface was matched to the reference surface, 10% of fractions had a residual setup error greater than their 5 mm tolerance.^10^ Setup errors were significantly reduced when the setup procedure combined surface imaging with kV/MV imaging correction.^10^

The above‐mentioned studies show that the use of surrogates to monitor the breath‐hold in DIBH is limited. Therefore, a way to directly QA the chest wall position would be beneficial. EPID images have been used to validate DIBH monitoring techniques.^8^, ^9^, ^17^ Conroy et al. manually measured the chest wall position on EPID images collected once a week to determine intrafraction motion, with manual measurements also made on each patient's DRR to calculate setup error.^8^ Lutz et al. analyzed EPID images taken during every third fraction using an in‐house method that required manual selection of the region of interest (ROI) to validate the RPM monitoring method.^9^ The resulting chest wall measurements were manually compared with the DRR to determine the setup error.^9^ Estoesta et al. also used manual measurements on EPID images collected every fifth fraction and DRR images to validate the laser‐tattoo technique for DIBH monitoring.^17^ Therefore, EPID images could be used as part of a QA process for DIBH without needing additional equipment. However, a tool to automatically review EPID images is needed, given the potentially substantial number of images. For example, with EPID images collected for analysis from each fraction the total number of images to review would range from 800 to 1250 for a typical course of treatment, making manual review of EPID images impractical.

Ideally, automated review should be done during patient treatment. However, the first step toward that goal consists of testing the feasibility of automated detection on retrospective images. This was the focus of this work. If the algorithms available to automatically review EPID images prove to be accurate, expansion of its use as an online tool could be explored. This would require a fast image‐processing algorithm capable of making chest wall measurements in real‐time, and a system to enable reading the EPID images as they are generated during treatment.^18^ This could also result in greater compliance with ACR‐ASTRO guidelines, which recommend that IGRT images are reviewed to verify correct dose delivery.^19^

Jensen et al. previously studied the automatic review of EPID images acquired during DIBH using a Canny filter to outline the chest wall.^20^ They collected these measurements for a ROI centered on the nipple position in the image and used their results to analyze intrafraction motion.^20^ Optimizing the Canny filter's user‐defined parameters is challenging when analyzing a large patient dataset. Hong et al. used a pattern‐matching algorithm to measure the horizontal and vertical changes in the chest wall and breast skin position to analyze the dosimetric impact of patient motion.^21^ Poulsen et al. implemented automated heart detection using a novel algorithm applied to EPID images from left‐sided DIBH breast treatments.^2^ Doebrich et al. located the chest wall position by measuring the pixel intensities of image rows and selecting the first maxima after the medial field edge.^22^ This algorithm required manually selecting superior‐inferior field edges for each fraction.^22^ Vasina et al. proposed an algorithm that used the EPID intensity profiles to measure the location of peak intensity within the breast as the chest wall.^18^ Their algorithm measures the chest wall position at three distinct levels, where each measurement is the average of three adjacent rows.^18^ Results from this algorithm were used to demonstrate that the external skin position may not correlate to the internal anatomical position of the chest wall.^18^ They note that using the peak intensity location may lead to greater uncertainty in the chest wall position for those patients or phantoms whose EPID image profiles exhibit a “broad peak.^18^”

This work presents a methodology that can be used to verify the patient position during the open tangents of the breast hybrid IMRT technique. Breast hybrid planning combines open tangents and IMRT fields to achieve the desired fluence.^23^ The open tangents and EPID images can be used to verify chest wall position during treatment. In this retrospective work, we compared the accuracy of different algorithms for measuring the chest wall position on EPID images from a dynamic thorax phantom and 20 left‐sided DIBH breast patients. One algorithm utilizes the Canny filter to outline the chest wall in each image, while the others analyzed image profiles and identified the chest wall as either a peak or the inflection point at the lung‐rib junction. The latter approach provides a physical reference point that can be used to validate the algorithm's results with manual measurements. Each algorithm's accuracy was quantified, and limitations were identified. Setup error and intrafraction motion were calculated for all fractions of 20 left‐sided breast patients. The static fields of a hybrid static‐IMRT technique were analyzed, with 10 of the patients having MLCs partially blocking their static fields, and 10 without MLCs. The fields partially blocked by MLCs corresponded to patients who received additional supraclavicular radiation, while the patients treated with fields without MLCs did not receive supraclavicular radiation. This work aims to select an algorithm for measuring the chest wall position in EPID images. This allows for the review of QA images to be automated and for direct monitoring of the chest wall without relying on an external surrogate. Such an algorithm could quantify intrafraction motion and setup error during breast RT.

## 2 METHODS

### 2.1 Breast hybrid planning

All patients were planned using External Beam Application in ARIA 15.6 (Varian Medical Systems, Palo Alto, California, USA) using a hybrid IMRT technique.^23^ This technique involves treating the patient with open tangential fields followed by IMRT tangential fields. The open fields are those static fields delivered without MLCs, defined by setting the jaws according to anatomical landmarks of clinical interest. Additional MLC shielding can be added to the open fields to shield the heart or to shield part of the lung when the collimator is set to zero degrees (for patients receiving supraclavicular irradiation). The jaws of the IMRT fields are set to the same size as the static fields, and the plan is optimized to deliver a uniform dose throughout the breast, typically resulting in a smooth sweep of MLCs. The static fields delivered between 50% and 80% of the dose, and these are the fields where EPID images were acquired and the position of the chest wall was identified. This was only done for the static fields since the sweeping MLCs of the IMRT fields will eventually block the chest wall.

### 2.2 DIBH treatment

All treatments were performed using the technique, which relies on the overlap of lasers to patient skin tattoos.^8^ Patient positioning was carried out using the reference tattoos from CT simulation and a marker line was drawn on the side of the patient to record their breath hold level. The patients were instructed to perform DIBH, while anterior portal images were taken to correct superior/inferior and left/right positioning errors. This was followed by another breath hold and a tangential portal image was acquired for setup to correct the remaining anterior/posterior positioning error within 3 mm. During beam delivery, the breath hold level was monitored by the radiation therapist which observed the overlap of the in‐room laser with the marker line drawn laterally on the patient. There were two open tangents and two IMRT tangents delivered in each fraction, with each beam requiring a separate breath hold. For each beam, the patient was instructed to hold their breath for up to 20 s while the beam was delivered. It should be noted that this technique does not rely on a quantitative gating window but on a qualitative assessment that laser and the tattoo overlap in order to ensure an acceptable breath. All EPID images analyzed in this study correspond to instances when the laser and tattoo were judged to be overlapping.

### 2.3 EPID images

EPID images were acquired using continuous acquisition mode on either a Clinac (Varian Medical Systems, Palo Alto, California, USA) or Trilogy (Varian Medical Systems, Palo Alto, California, USA) linear accelerator. *Cine* images were collected from both the medial and lateral tangential fields of each fraction with either a 6 or 23 MV (12 of the 20 patients had images made with 23 MV beams) treatment beam at 600 MU/min. Beam delivery ranged from 10 to 26 s with the number of cine images per beam being 15 on average. Images were manually exported in DICOM format from Offline Review (Varian Medical Systems, Palo Alto, California, USA).

### 2.4 Algorithms

**FIGURE 1:**
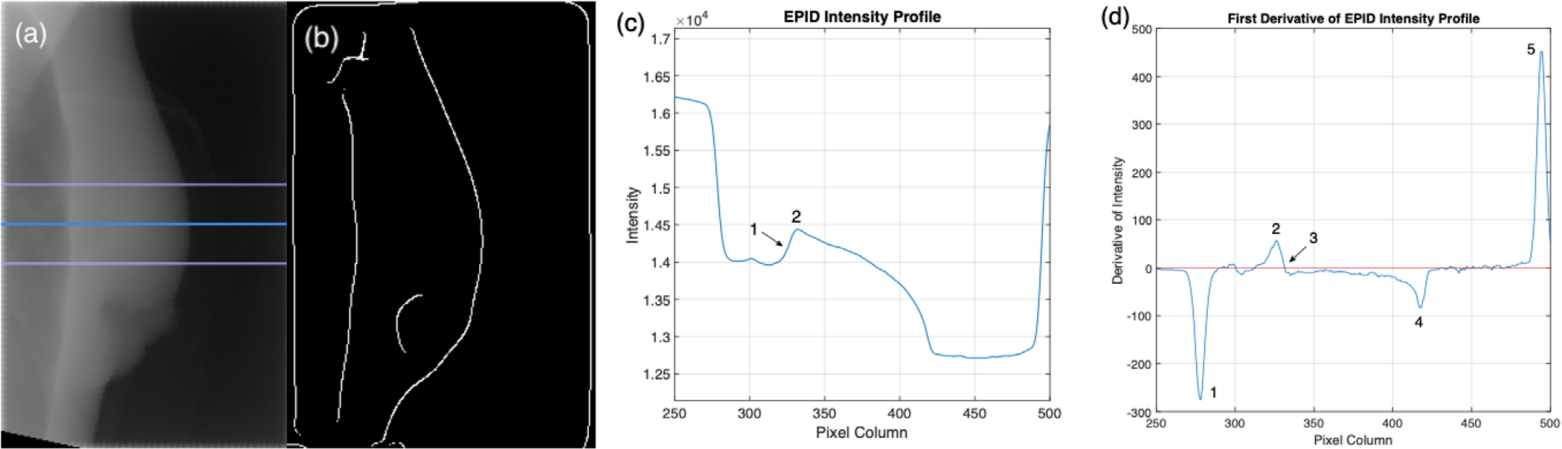
EPID images and profiles. (a) EPID image of the tangential field. The top and bottom blue lines indicate the upper and lower bounds of the region of interest, calculated to span 1.5 cm above and below the image center, indicated by the center line. (b) After applying the Canny filter, the corresponding image shows edges thinned to a single‐pixel thickness. (c) Intensity profile demonstrating the method of the peak‐intensity algorithm. The steep slopes on either end of the profile indicate the medial and lateral borders of the radiation field. Index 2 shows the peak intensity within the breast. (d) The first derivative of the intensity profile from (c), used by the inflection‐point algorithm. Index 1 shows the medial field border. The first derivative maxima within the breast, corresponding to the inflection point (Index 1 on Figure 1c), is indicated at index 2. The zero‐crossing point corresponding to the peak intensity in the breast (Index 2 on Figure 1c) is indicated at index 3, with the zero‐line highlighted in red. The drop in attenuation at the skin‐air interface is at index 4, and the lateral field border is at index 5.

Three different algorithms were tested to evaluate their accuracy in identifying the chest wall's location. All algorithms were developed using commercially available software (MATLAB, MathWorks, R2015b). The first algorithm was based on work done by Jensen et al. to monitor intrafraction motion during DIBH breast treatments.^20^ This work used the Canny filter to outline the chest wall in each EPID image, as seen in Figure 1b. The Canny filter requires two inputs from the user. The first is the sigma value of the Gaussian smoothing filter, which removes image noise before edge detection is applied. The second parameter controls the thresholding of the Canny filter, determining which edges are strong enough to be preserved by the edge detection process.^24^ Both parameters were adjusted for phantom and patient images as described below, and a commercial implementation of the Canny filter was used (MATLAB, MathWorks, R2015b).

The second algorithm was developed in‐house^25^ and was also developed independently and in parallel by Doebrich et al.^22^ and Vasina et al.^18^ This algorithm analyses the pixel intensity profiles of the EPID frames and finds the location of peak intensity within the breast. The chest wall position is defined as the distance from the medial border to the peak intensity location. The algorithm developed in‐house records this distance along a 61‐row ROI (the midline with 30 rows above and below) centered halfway along the superior‐inferior direction (Figure 1a).^25^ The median distance is recorded as the chest wall position in each frame with the standard deviation as the uncertainty.^25^ Doebrich et al. measured this distance along the midline profile,^22^ with Vasina et al. reporting this distance at three equidistant profiles in each frame.^18^

**FIGURE 2:**
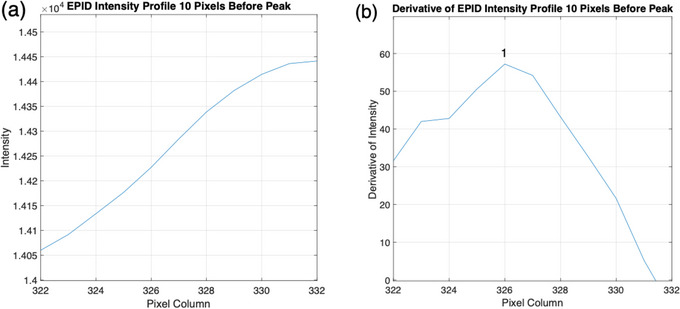
Inflection point calculation. (a) EPID intensity profile cropped to represent the 10 pixels leading up to the peak intensity. (b) First derivative of the cropped profile. Index 1 shows the maxima of the first derivative which indicates the location of the inflection point at the lung‐rib junction.

The third algorithm is a modified version of the previous algorithm, where the inflection point at the lung‐rib junction is chosen as the chest wall instead of the peak intensity (Figure 1c). It was hypothesized that this would provide better agreement with the end of the lung tissue, which is used for manual review of chest wall position. In order for intrafraction motion and setup error results to be manually validated, the chest wall measurements made by the algorithm need to correspond to an anatomical landmark visible to the reviewer in Offline Review. The brightest pixel within the rib region calculated by the second (peak intensity) algorithm is not suitable for this purpose. This is because the peak intensity can fall anywhere within the rib and may not directly correlate with manual measurement performed by an observer in Offline Review. To find the inflection point at the lung‐rib junction, the location of peak intensity within the breast is first found. Then the profile is cropped to include only the 5 mm leading up to this peak to isolate the section of the profile containing the end of the lung tissue and the beginning of the rib bone (Figure 2a). This distance of 5 mm was chosen to account for the thickness of the rib bone, which was observed to be as high as 5 mm in Offline Review. The first derivative of the cropped profile is then calculated, with the maxima indicating the location of the inflection point (Figure 2b). Like the previous algorithms, the distance between the medial border and the inflection point is recorded for a 61‐row ROI with the median and standard deviation calculated. The use of this algorithm for chest wall detection therefore has the advantage over the second algorithm of being able to be manually validated more accurately (validation of the peak intensity algorithm is limited to the rib thickness) while also being less dependent on the choice of parameters as the Canny algorithm.

### 2.5 Phantom validation

**FIGURE 3:**
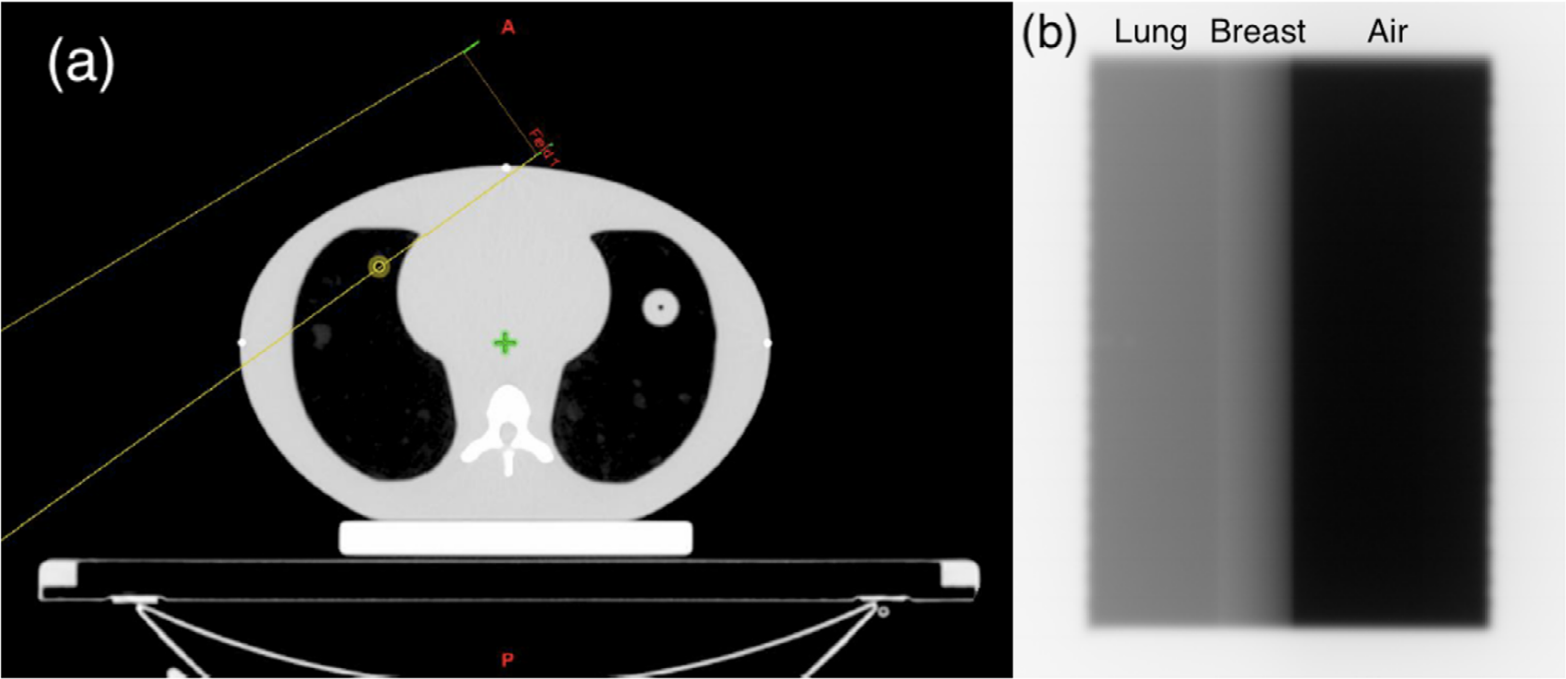
Dynamic thorax phantom. (a) Schematic diagram of dynamic thorax phantom, with the yellow outline showing radiation field boundaries. (b) Sample EPID image from thorax phantom with different tissue types labeled. The amount of exposed lung was varied by adjusting the MLC position in increments of 1, 2, 5, and 10 mm.

Phantom studies were used to assess the algorithms’ ability to measure the chest wall position against known changes in lung tissue. The three algorithms were applied to EPID images from a dynamic thorax phantom (Sun Nuclear, Model 008A) shown in Figure 3. The thorax phantom was kept static during irradiation and the amount of exposed lung tissue was changed by adjusting the MLC position. Four EPID images were analyzed where the amount of exposed lung tissue equaled 1, 2, 5, and 10 mm, respectively. The Canny filter parameters for thresholding and smoothing of the phantom images were based on work from Jensen^20^ and calibrated for the phantom dataset to have an upper threshold of 0.02, lower threshold of 0.01, and a sigma value of 2.

### 2.6 Validation with patient images

Patient images from left‐sided breast treatments were used to test the accuracy of the algorithms in measuring the chest wall across the changes in anatomy seen in a sample of 10 patients. The first EPID frame from each of the 10 patients treated that did not receive supraclavicular radiation (without MLCs in the field) was compared against manual measurements taken in Offline Review (Varian Medical Systems, Palo Alto, California, USA). The lack of MLCs in the field made it easier to validate the algorithms at an early stage (analysis of images with MLCs is described below). The EPID images had their window level adjusted to increase the contrast of the lung and breast tissue. The ROI was measured to span 3 cm, centered halfway between the superior and inferior image borders. For each frame, 10 manual measurements were made across the ROI from the medial border to the end of the lung with the distance tool in Offline Review. The median was taken as the result with the standard deviation as the uncertainty. These were compared to the results from all three algorithms.

The user‐defined parameters of the Canny filter were optimized for the 10‐patient dataset. This was performed by testing the Canny filter on the patients' first EPID images while varying the smoothing parameter from 1 to 10 and the thresholding parameter from 0.001 to 0.02. The algorithm results for each image were compared to the median of 10 manual measurements of the same ROI conducted in Offline Review to find the optimal Canny parameters.

For those patients treated with MLCs present in the field, there are concerns associated with measuring the chest wall position from the medial field edge. Patient motion in the superior‐inferior direction, together with changing MLC shielding, can affect the amount of lung exposed within the ROI, altering the distance between the medial field edge (given by MLC position) and the chest wall. This may result in the appearance of intrafraction motion in the anterior‐posterior direction. To remedy this, all intrafraction motion and setup error data were obtained from chest wall measurements made from the lateral field edge.

### 2.7 Intrafraction and setup measurements

**FIGURE 4:**
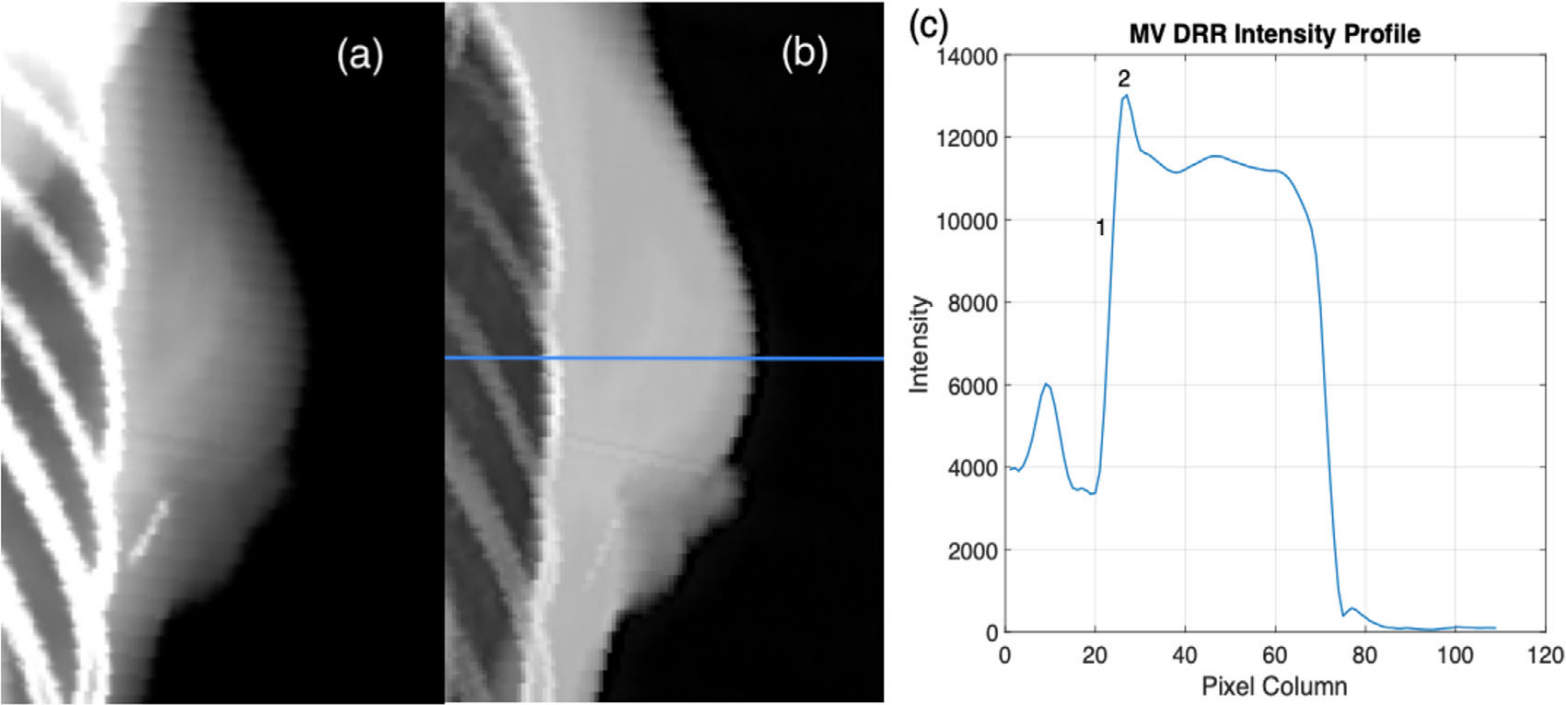
kV and MV DRR comparison. (a) kV DRR image showing the contrast between the ribs and lung tissue. (b) MV DRR image created by adjusting the window level to reduce the relative intensity of bone, such that the intensity profile will share the same peak within the breast as MV portal images. (c) MV DRR intensity profile along the central axis. Indexes 1 and 2 represent the positions of the inflection point at the lung‐rib junction and the peak intensity within the breast, respectively. The inflection point at Index 1 is marked as the chest wall position for each row in the region of interest.

The inflection‐point algorithm was used to measure intrafraction motion along with the accuracy of setup for DIBH patients. Intrafraction motion was measured as the change in chest wall position in each subsequent EPID frame with respect to the first EPID frame of a field. These results were recorded for all fractions of 20 left breast patients' treatments, and we report the maximum motion found per beam. To measure the setup error, the parameters of each DRR generated with the treatment planning system were adjusted to simulate a MV image so that the same algorithm could be applied to the DRR as the EPID images. The window level was adjusted such that CT numbers ranging from −1000 to 100 were given a relative weight of 0.5, and CT numbers from 100 to 1000 were given a weighting of 2.0. The profile of the MV DRR shares the peak associated with the rib bone, shown in Figure 4, which allows for this algorithm to also identify the chest wall as the inflection point at the end of the lung tissue. As in the EPID images, the algorithm searches for an upward inflection point within 5 mm leading up to the peak intensity to locate the lung‐rib interface. In order to cover the same 3 cm range as the 61‐pixel ROI in the EPID images, a 31‐row ROI was used on each DRR. Again, the median distance calculated in this ROI is taken as the chest wall location, with the standard deviation recorded as the uncertainty. The inflection‐point algorithm was validated for MV DRR images by comparing the chest wall measurements from the MV DRR images of the 10‐patient dataset with manual measurements conducted in Offline Review. These manual measurements were taken from the medial field border to the end of lung tissue on the kV DRR in Offline Review. The setup error was calculated as the difference between the chest wall positions calculated for the DRR and the first frame of the EPID images. The largest positive and negative setup errors for the non‐supraclavicular and supraclavicular patient groups were compared against manual measurements in Offline Review.

## 3 RESULTS

### 3.1 Phantom measurements

**FIGURE 5:**
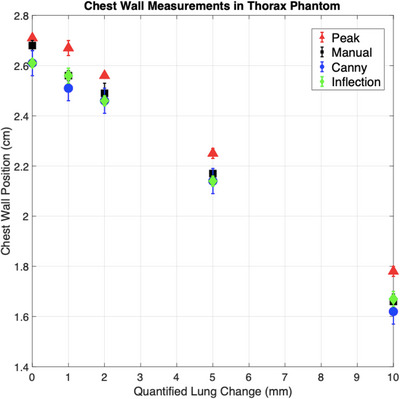
Algorithm results on dynamic thorax phantom. Chest wall measurements from the Canny, inflection‐point, and peak‐finding algorithms on EPID images taken from the thorax phantom. The amount of exposed lung was changed in increments of 1, 2, 5, and 10 mm by adjusting the multi‐leaf collimator position. The inflection‐point algorithm had the closest agreement with manual measurements, at a mean difference of 0.3 mm.

Figure 5 shows the chest wall positions calculated by the Canny algorithm, peak‐finding algorithm, and manual measurements for the thorax phantom. The inflection point algorithm had the closest agreement with manual measurements, with a mean difference of 0.3 mm. The Canny and peak‐finding algorithms had mean agreements of 0.4  and 0.8 mm, respectively. The largest difference between a manual and algorithm measurement was 1.2 mm, with the biggest difference between the three algorithms' measurements being 1.6 mm. The largest uncertainty was 0.3 mm for both the inflection algorithm and peak‐finding algorithm, while the Canny algorithm's largest uncertainty was 0.2 mm. The largest uncertainty was 0.4 mm for the manual measurements.

### 3.2 Algorithm validation with patient images

Table 1 shows the chest wall positions calculated by all algorithms and manual measurements for the first frame from each of the 10 patients. The set of Canny parameters yielding the highest mean agreement with manual measurements was chosen as the optimal parameters. This resulted in a threshold parameter of 0.006 and Gaussian smoothing with a sigma value of 4 yielding the highest agreement (2.0 mm with a standard deviation of 0.6 mm for the 10‐patient dataset). To the authors’ knowledge, there currently is no standard method for optimizing the Canny filter parameters for EPID images. These customized set of parameters allowed us to benchmark the accuracy of the Canny algorithm as a best‐case scenario for this group of patients and may need to be modified for other patients.

**TABLE 1:** Algorithm chest wall data compared to manual measurements for EPID images

Patient	Canny algorithm (cm)	Peak‐finding algorithm (cm)	Inflection‐point algorithm (cm)	Manual measurement (cm)
P1	2.48± 0.04	2.88± 0.07	2.58± 0.09	2.61± 0.07
P2	2.30± 0.03	2.70± 0.04	2.45± 0.04	2.54± 0.03
P3	2.01± 0.02	2.35± 0.17	2.11± 0.16	2.19± 0.06
P4	1.62± 0.04	1.91± 0.04	1.72± 0.05	1.74± 0.04
P5	1.81± 0.34	2.30± 0.07	1.96± 0.10	2.14± 0.06
P6	2.30± 0.07	2.65± 0.05	2.45± 0.06	2.48± 0.08
P7	1.32± 0.06	1.67± 0.06	1.47± 0.08	1.50± 0.09
P8	2.35± 0.59	2.74± 0.07	2.50± 0.06	2.56± 0.08
P9	1.91± 0.33	2.30± 0.08	2.06± 0.06	2.12± 0.04
P10	1.96± 0.06	2.30± 0.04	2.06± 0.11	2.17± 0.06

Chest wall measurements from the three different image‐processing algorithms compared to manual measurements for the 10‐patient dataset. The first algorithm used the Canny filter to outline the chest wall, while the other two used either the peak position within the breast or the inflection point leading up to the peak as the chest wall position. For each algorithm, the median chest wall position within the region of interest was compared to the median of ten manual measurements within the same region. Uncertainties for both the algorithm and manual measurements are given by the standard deviation of chest wall measurements within the region of interest.

The peak‐finding algorithm's results were up to 2.7 mm larger than those obtained by manual measurements (Table 2). We hypothesize that this was caused by the peak intensity occurring inside the bony rib portion of the image, while the manual measurements were taken at the end of the lung tissue in the EPID images. The inflection‐point algorithm had the closest agreement with manual measurements, at 0.7 ± 0.5 mm on average for the 10‐patient dataset. Thus, the inflection‐point algorithm was chosen for subsequent analysis of intrafraction motion and setup accuracy. The Canny algorithm had the largest uncertainties, which was due to noise within the lung region interfering with the chest wall measurements.

**TABLE 2:** Summarized results of algorithm validation for EPID images

Algorithm	Mean agreement (mm)	Standard deviation (mm)	Max difference (mm)
Canny	2.0	0.6	3.3
Peak‐finding	1.8	0.4	2.7
Inflection‐point	0.7	0.5	1.8

For the 10‐patient dataset, the mean agreement, standard deviation of agreement, and maximum disagreement were recorded for each of the three algorithms. The inflection‐point algorithm had the closest mean agreement to manual measurements and the smallest maximum difference and was chosen for subsequent analysis of intrafraction motion and setup errors.

### 3.3 Algorithm validation with MV DRR images

Table 3 shows the validation results regarding the ability of the inflection‐point algorithm to identify the chest wall on MV DRR. Table 3 also lists the results of manual measurements done on kV DRR images. The inflection‐point algorithm had a mean agreement of 1.2 mm and an uncertainty of 1.0 mm. The largest recorded disagreement was 3.3 mm, and the smallest was 0.4 mm. The DRR images were of lower resolution than the EPID images, as they are composed of roughly half as many pixels, which limited the accuracy of chest wall detection. The relatively high standard deviations of these measurements reflect the curvature of the rib bones and their overlapping positions within the ROI.

**TABLE 3:** Validation of inflection‐point algorithm on MV DRR images

Patient	Inflection‐point algorithm (cm)	Manual measurement (cm)	Difference (mm)
P1	2.25 ± 0.08	2.20 ± 0.14	0.5
P2	2.34 ± 0.08	2.38 ± 0.17	0.4
P3	1.90 ± 0.12	1.94 ± 0.14	0.4
P4	1.86 ± 0.06	1.91 ± 0.35	0.5
P5	1.95 ± 0.07	1.74 ± 0.38	2.1
P6	2.05 ± 0.13	1.97 ± 0.22	0.8
P7	1.66 ± 0.19	1.62 ± 0.34	0.4
P8	2.44 ± 0.07	2.11 ± 0.47	3.3
P9	1.95 ± 0.12	1.78 ± 0.24	1.7
P10	1.95 ± 0.05	1.81 ± 0.45	1.4

Results of the inflection‐point algorithm validation. The median chest wall position in a 31‐row region of interest was recorded with the median measurement obtained from 10 patients' MV DRR images. These were compared to the median of 10 manual measurements taken from the same region on the 10 patients' kV DRR images in Offline Review.

### 3.4 Intrafraction motion

**FIGURE 6:**
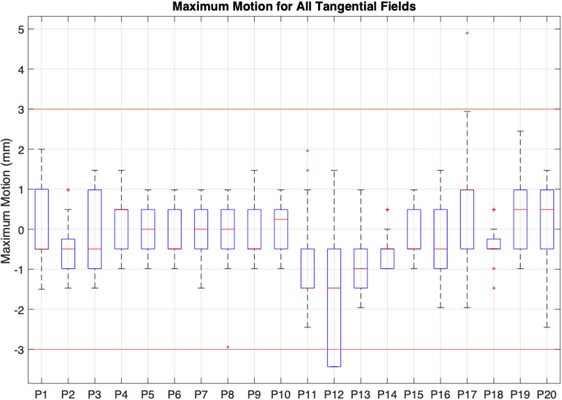
Intrafraction motion results for 20‐patient cohort. The maximum motion per beam was recorded and displayed in boxplots for all patients. The largest motion was recorded as +4.9 mm, outside of the 3 mm tolerance indicated by the red horizontal lines.

Figure 6 shows boxplots of the maximum intrafraction motion calculated in each beam for the 20‐patient cohort. On average, the maximum motion was −0.2 mm. The maximum calculated motion in the positive direction was a +4.9 mm shift occurring in P17. This measurement was manually validated as +4.1 mm, and the motion was such that the heart could be seen in the EPID image while it was absent at the beginning of the treatment. The maximum motion in the negative direction was −3.4 mm, which occurred in 16 beams of P12. The first of these measurements was manually validated as −2.2 mm. The maximum intrafraction motion was within the 3 mm tolerance level for 98% of the delivered fields.

### 3.5 Setup error

**FIGURE 7:**
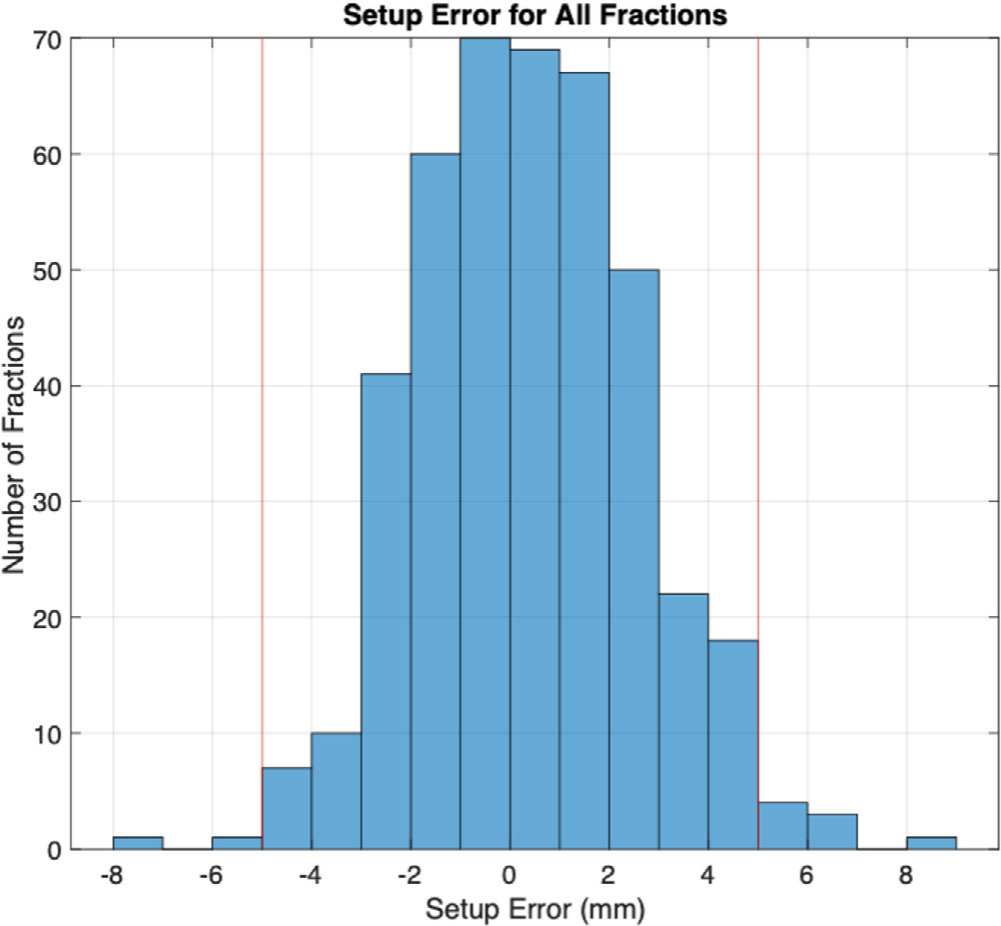
Setup error results for 20‐patient cohort. The difference in chest wall position between the first EPID image of the lateral beam and the MV DRR is displayed for all patient fractions. Positive setup errors represent a deeper breath hold at treatment than simulation, and negative errors represent a shallower breath hold at treatment.

Figure 7 shows histograms of the setup errors for all fractions. The mean setup error was +0.3 mm. The maximum negative setup error was −7.1 mm, which was manually validated as −5.7 mm. The maximum positive setup error of +8.9 mm was manually validated as +8.5 mm. About 97.6% of the fractions had a calculated setup error within our 5 mm tolerance level, with 10 fractions outside tolerance.

## 4 DISCUSSION

This work describes a methodology to automatically find the chest wall during irradiation of the open fields for a breast hybrid IMRT technique. The accuracies of three algorithms were compared in EPID images from left‐sided breast patients against manual measurements (the current standard of practice). The closest agreement with manual measurements achieved with the Canny algorithm was 2.0 ± 0.6 mm. A difficulty with the Canny filter is that smoothing was seen to cause distortions in the patient anatomy. Gaussian smoothing is required for noise reduction prior to edge detection, but this parameter should be minimized to limit the distortion of the chest wall outline.

Additionally, since the Canny filter produces a binary image with all edges thinned to a single pixel thickness, the only way to distinguish between the different features of the Canny image (medial border, chest wall, breast outline, etc.) is based on the relative position of the edges. If there is any noise within the lung region, these pixels will be misidentified as the chest wall.

The peak‐finding algorithm had the second largest disagreement with manual measurements since the peak can occur anywhere within the patient's rib, and rib thickness was observed to be up to 5 mm. Note that this algorithm had a higher agreement with manual measurements in the results of the phantom measurements since no rib is present. It should be noted that the peak‐finding algorithm may still be used to quantify intrafraction motion as long as the relative position of the peak remains constant with respect to the patient anatomy. The peak position is a function of patient anatomy and geometry (which, in turn, is a function of patient setup). The inflection‐point algorithm only depends on the patient anatomy and gave the closest agreement with manual measurements (the current standard at our center). The inflection‐point algorithm was therefore chosen to quantify intrafraction motion and setup accuracy.

The methods presented in this study are currently limited to the open fields of the hybrid IMRT approach. This approach typically consists of two open fields and two IMRT fields. In this study, only EPID images from the open fields were analyzed. This leaves the possibility of patient motion going undetected during the IMRT portions of the treatment. Future work could address this issue by finding a way to identify the chest wall position during the IMRT field, incorporating the information of MLC control points.

**FIGURE 8:**
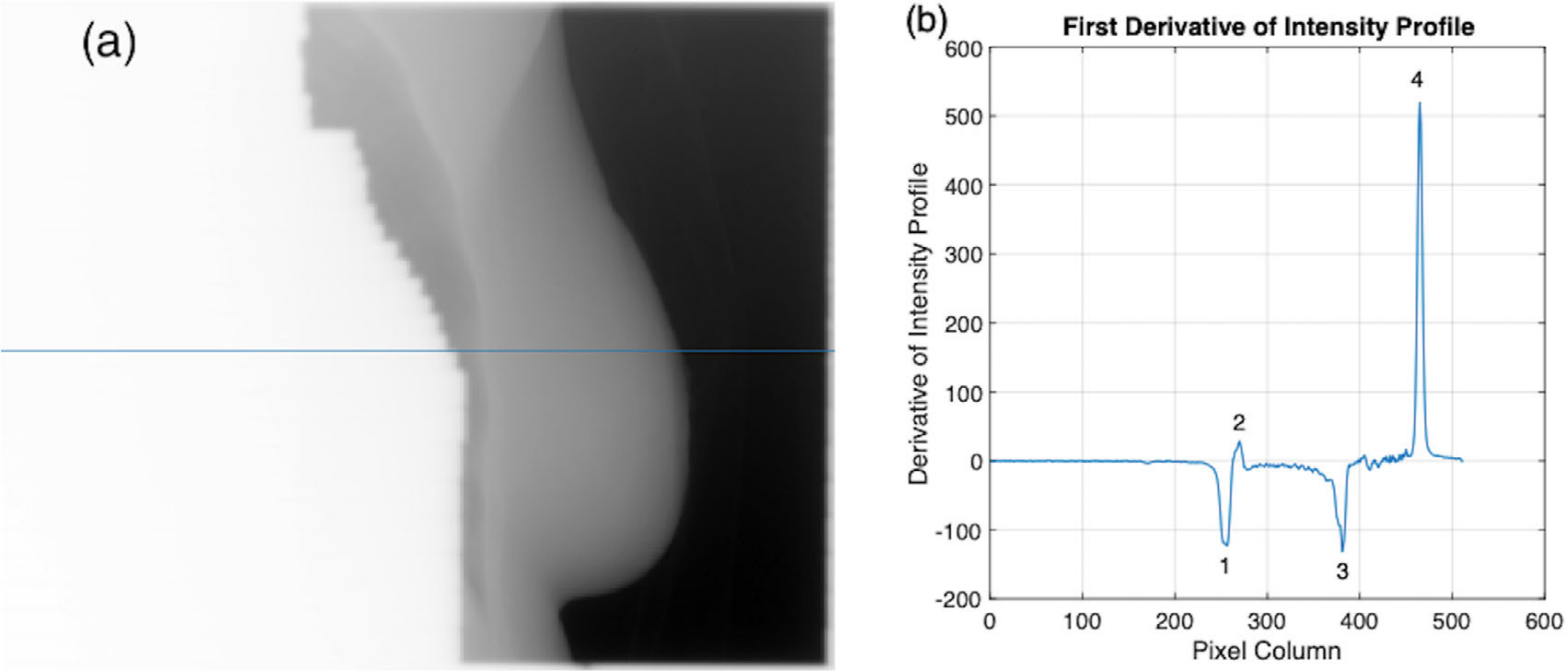
Image and profile showing incorrect cropping of EPID profile. (a) EPID image from tangential breast field, the blue line indicates the pixel row where the gradient at the skin‐air interface is steeper than that of the medial field border. (b) Derivative of EPID intensity profile at the pixel row indicated in (a). The gradient at the medial border, indicated by index 1, is less extreme than the gradient at the skin‐air interface shown at index 3. This was hypothesized to be caused by a rib bone occupying the short lung region exposed in this row, which interfered with the algorithm's detection of the medial border, defined as the point of greatest negative gradient. The positive gradients at indices 2 and 4 represent the inflection point at the chest wall and the transition from air to the lateral field border, respectively.

For one patient in this study, it was seen that the medial field border was misidentified due to the MLC extending nearly to the bony chest wall for some rows within the ROI. These images were found to be incompatible with the inflection‐point algorithm. It was hypothesized that a rib bone adjacent to the MLC caused a reduced gradient value in that area (Figure 8). Since the medial field border is defined as the steepest decline in intensity across the profile, it was incorrectly detected as the breast‐air interface in the profile below. This resulted in incorrect cropping of the profile and incorrect measurements. A proposed solution to this issue was implemented such that if the distance between the detected medial and lateral field edges was less than 5.5 cm, the second‐steepest intensity decline across the profile was set as the medial border. This value of 5.5 cm was chosen since the smallest tangential field in the available EPID database was 6.02 ±  0.1 cm at its narrowest point. A 5 mm margin was subtracted from this distance to result in 5.5 cm. This adjustment yielded accurate measurements for this patient's EPID images, but additional EPID images with similar MLC shielding are needed to test the proposed correction.

An additional limitation of the current version of the algorithm is the presence of bolus. Bolus can result in air gaps that can potentially lead to an incorrect location of the skin‐air interface. Therefore, patients with bolus were excluded from this study. Finally, it should be noted that some images had to be removed from the analysis due to the improper placement of the EPID device during treatment. It is necessary that the full “flash” component be captured, such that the image profile contains a steep incline to represent the lateral border and allow for correct cropping of the profile. This resulted in EPID images from 6 beams being excluded from the analysis. A consistent procedure for EPID positioning would help alleviate this issue.

Of the 20 patients whose images were analyzed in this study, eight were treated with 6‐MV beams and 12 with 23‐MV beams. All three algorithms were able to detect the chest wall position on images of both energies. The relationship between beam energy and the accuracy of the inflection‐point algorithm was beyond the scope of this study, but could be explored in future work.

The images from this study were generated using C‐series linear accelerators (Varian Medical Systems, Palo Alto, California, USA) equipped with the aS1000 model EPID. *Cine* images were saved in DICOM format and the algorithms extracted information such as the gantry angle, collimator angle, and jaw positions from the DICOM header. TrueBeam systems can be fitted with the same EPID model or the aS1200 model. The main differences between these models are that the aS1200 model features a larger active area and smaller pixel sizes than the aS1000 (due to an increased number of pixels). Given the smaller pixel sizes of the aS1200 model, the accuracy of chest wall detection may be enhanced, but this requires further investigation. Another challenge that may need to be overcome is that TrueBeam systems save *cine* images in MPEG format instead of DICOM. Without the DICOM header, the algorithms would have to be modified to extract the needed information from a different source (e.g., directly from the treatment plan).

## 5 CONCLUSION

A methodology has been developed to automatically calculate the location of the chest wall in EPID images acquired during DIBH treatments planned with a hybrid IMRT approach. Three algorithms were tested on their ability to measure the chest wall in EPID images taken from a dynamic thorax phantom and a 10‐patient dataset. The inflection‐point algorithm had the closest agreement with manual measurements, with a mean difference of 0.7 mm. When tested with MV DRR images, this algorithm had a mean agreement of 1.2 mm. Furthermore, it measures intrafraction motion and setup errors within 1.4 mm of manual measurements. Future work includes analyzing the data of more patients and testing the algorithm on the IMRT portions of DIBH breast treatments.

## AUTHOR CONTRIBUTIONS

Jonathan Redekopp performed acquisition and analysis of data, contributed to design of algorithms, and produced the written manuscript with Jorge Alpuche. Jorge Alpuche, Ryan Rivest, and David Sasaki contributed to acquisition of data and the conception of the work in implementing DIBH at our center. Jorge Alpuche and Ryan Rivest also contributed to design of algorithms. Stephen Pistorius aided in the interpretation and analysis of data. All authors reviewed and gave final approval of the written manuscript. All authors agree to be responsible for the accuracy and integrity of results contained within the manuscript.

## CONFLICT OF INTEREST STATEMENT

No conflict of interest.
